# 4-[(*E*)-(2-Methoxy­phen­yl)imino­meth­yl]-*N*,*N*-dimethyl­aniline

**DOI:** 10.1107/S1600536810017228

**Published:** 2010-05-15

**Authors:** Shan-Bin Liu, Cai-Feng Bi, Qiang Wang, Jian Zuo, Yu-Hua Fan

**Affiliations:** a Key Laboratory of Marine Chemistry Theory and Technology, Ministry of Education, College of Chemistry and Chemical Engineering, Ocean University of China, Qingdao, Shandong 266100, People’s Republic of China

## Abstract

In the title compound, C_16_H_18_N_2_O, the dihedral angle between the benzene rings is 38.5 (2)°. The crystal packing is stabilized by weak C—H⋯N and C—H⋯O inter­actions and aromatic π–π stacking [centroid–centroid separations = 3.620 (5) and 3.546 (4) Å].

## Related literature

For general background to Schiff bases, see: Atwood & Harvey (2001 ▶). For a related structure, see: Liu *et al.* (2009 ▶).
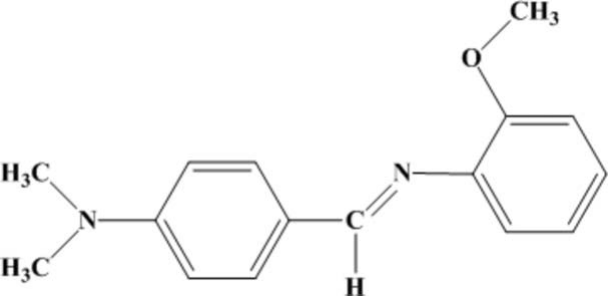


## Experimental

### 

#### Crystal data

C_16_H_18_N_2_O
*M* *_r_* = 254.32
Orthorhombic, 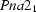

*a* = 15.182 (8) Å
*b* = 11.756 (6) Å
*c* = 7.809 (4) Å
*V* = 1393.8 (13) Å^3^
*Z* = 4
Mo *K*α radiation
μ = 0.08 mm^−1^
*T* = 298 K
0.60 × 0.58 × 0.49 mm

#### Data collection

Siemens SMART CCD diffractometer
Absorption correction: multi-scan (*SADABS*; Siemens, 1996 ▶) *T*_min_ = 0.956, *T*_max_ = 0.963
6900 measured reflections
2335 independent reflections
1554 reflections with *I* > 2σ(*I*)
*R*_int_ = 0.055

#### Refinement

*R*[*F* ^2^ > 2σ(*F* ^2^)] = 0.043
*wR*(*F* ^2^) = 0.123
*S* = 1.01
2335 reflections
172 parameters
1 restraint
H-atom parameters constrained
Δρ_max_ = 0.16 e Å^−3^
Δρ_min_ = −0.18 e Å^−3^

### 

Data collection: *SMART* (Siemens, 1996 ▶); cell refinement: *SAINT* (Siemens, 1996 ▶); data reduction: *SAINT*; program(s) used to solve structure: *SHELXS97* (Sheldrick, 2008 ▶); program(s) used to refine structure: *SHELXL97* (Sheldrick, 2008 ▶); molecular graphics: *SHELXTL* (Sheldrick, 2008 ▶); software used to prepare material for publication: *SHELXTL*.

## Supplementary Material

Crystal structure: contains datablocks global, I. DOI: 10.1107/S1600536810017228/hb5420sup1.cif

Structure factors: contains datablocks I. DOI: 10.1107/S1600536810017228/hb5420Isup2.hkl

Additional supplementary materials:  crystallographic information; 3D view; checkCIF report

## Figures and Tables

**Table 1:** Hydrogen-bond geometry (Å, °)

*D*—H⋯*A*	*D*—H	H⋯*A*	*D*⋯*A*	*D*—H⋯*A*
C8—H8*C*⋯N1^i^	0.96	2.67	3.620 (5)	170
C4—H4⋯O1^i^	0.93	2.64	3.546 (4)	166

Symmetry code: (i) 
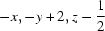
.

**Fig. 1.**
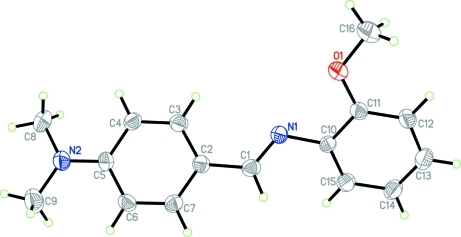
The structure of (I) showing 30% probability displacement ellipsoids.

**Fig. 2.**
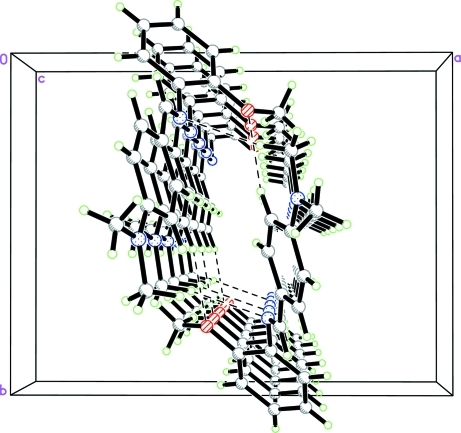
A view of the crystal structure of (I) showing chain to the c linked via C—H···N and C—H···O contacts.

## supplementary crystallographic information

### Comment

Schiff base ligands are among the most fundamental chelating systems in coordination chemistry (e.g. Atwood & Harvey, 2001). Herein, we present the synthesis and structure of a new schiff base ligand, 4-{(*E*)-[(2-methoxyphenyl)imino]methyl}-*N*,*N*-dimethylaniline.

The crystal structure of the title compound is given in Fig. 1. The bond lengths and angles (Table 1) in the title compound are found to have normal values (Liu *et al.*, 2009). This compound has a non-planar molecular structure, the dihedral angle between the two benzene rings is 38.54 °. In the crystal, the adjacent molecules are stabilized by non-classical C—H···N and C—H···O hydrogen bonding, with the distance of 3.620 (5) and 3.546 (4) Å (Table 2), respectively. Molecules are linked into chain along the c axis by the above weak interactions (Fig. 2).

### Experimental

4-(dimethylamino) benzaldehyde (10 mmol, 1.492 g) was added with stirring to anhydrous ethanol (30 ml) and an anhydrous ethanol solution (10 ml) of 2-methoxybenzenamine (10 mmol, 1.232 g) was slowly added. The reaction mixture was stirred at 353 K for 4 h, a yellow solid then separated out. The precipitate formed was filtered off, washed several times with anhydrous ethanol and dried under vacuum. Yellow blocks of (I) were obtained from anhydrous ethanol solution after 10 days by slow evaporation at room temperature.

### Refinement

The absolute structure of (I) is indeterminate based on the present refinement. All H-atoms were positioned geometrically and refined using a riding model, with C—H = 0.96 Å (methyl), 0.93 Å (methenyl), 0.93 Å (aromatic), and *U*_iso_(H) =1.2*U*_eq_(C).

### Crystal data

**Table:**

C_16_H_18_N_2_O	*F*(000) = 544
*M**_r_* = 254.32	*D*_x_ = 1.212 Mg m^−^^3^
Orthorhombic, *P**n**a*2_1_	Mo *K*α radiation, λ = 0.71073 Å
Hall symbol: P 2c -2n	Cell parameters from 1616 reflections
*a* = 15.182 (8) Å	θ = 2.7–21.5°
*b* = 11.756 (6) Å	µ = 0.08 mm^−^^1^
*c* = 7.809 (4) Å	*T* = 298 K
*V* = 1393.8 (13) Å^3^	Block, yellow
*Z* = 4	0.60 × 0.58 × 0.49 mm

### Data collection

**Table:**

Siemens SMART CCD diffractometer	2335 independent reflections
Radiation source: fine-focus sealed tube	1554 reflections with *I* > 2σ(*I*)
graphite	*R*_int_ = 0.055
φ and ω scans	θ_max_ = 25.0°, θ_min_ = 2.2°
Absorption correction: multi-scan (*SADABS*; Siemens, 1996)	*h* = −17→18
*T*_min_ = 0.956, *T*_max_ = 0.963	*k* = −13→11
6900 measured reflections	*l* = −9→9

### Refinement

**Table:**

Refinement on *F*^2^	Primary atom site location: structure-invariant direct methods
Least-squares matrix: full	Secondary atom site location: difference Fourier map
*R*[*F*^2^ > 2σ(*F*^2^)] = 0.043	Hydrogen site location: inferred from neighbouring sites
*wR*(*F*^2^) = 0.123	H-atom parameters constrained
*S* = 1.01	*w* = 1/[σ^2^(*F*_o_^2^) + (0.0524*P*)^2^ + 0.2535*P*] where *P* = (*F*_o_^2^ + 2*F*_c_^2^)/3
2335 reflections	(Δ/σ)_max_ < 0.001
172 parameters	Δρ_max_ = 0.16 e Å^−^^3^
1 restraint	Δρ_min_ = −0.17 e Å^−^^3^

### Special details

**Table:**

Geometry. All esds (except the esd in the dihedral angle between two l.s. planes) are estimated using the full covariance matrix. The cell esds are taken into account individually in the estimation of esds in distances, angles and torsion angles; correlations between esds in cell parameters are only used when they are defined by crystal symmetry. An approximate (isotropic) treatment of cell esds is used for estimating esds involving l.s. planes.
Refinement. Refinement of *F*^2^ against ALL reflections. The weighted *R*-factor wR and goodness of fit *S* are based on *F*^2^, conventional *R*-factors *R* are based on *F*, with *F* set to zero for negative *F*^2^. The threshold expression of *F*^2^ > σ(*F*^2^) is used only for calculating *R*-factors(gt) etc. and is not relevant to the choice of reflections for refinement. *R*-factors based on *F*^2^ are statistically about twice as large as those based on *F*, and *R*- factors based on ALL data will be even larger.

### Fractional atomic coordinates and isotropic or equivalent isotropic displacement parameters (Å^2^)

**Table:**

	*x*	*y*	*z*	*U*_iso_*/*U*_eq_	
N1	0.08529 (16)	0.7420 (2)	1.1920 (4)	0.0457 (6)	
N2	0.1439 (2)	1.0507 (2)	0.5158 (4)	0.0597 (8)	
O1	−0.04554 (14)	0.71462 (18)	1.4179 (3)	0.0612 (6)	
C1	0.10910 (19)	0.7230 (3)	1.0385 (5)	0.0483 (8)	
H1	0.1249	0.6490	1.0095	0.058*	
C2	0.11330 (18)	0.8091 (2)	0.9052 (4)	0.0441 (7)	
C3	0.0861 (2)	0.9208 (2)	0.9266 (5)	0.0498 (8)	
H3	0.0622	0.9424	1.0313	0.060*	
C4	0.0930 (2)	1.0008 (3)	0.7990 (4)	0.0488 (8)	
H4	0.0721	1.0742	0.8172	0.059*	
C5	0.1316 (2)	0.9720 (3)	0.6405 (4)	0.0441 (8)	
C6	0.1559 (2)	0.8582 (3)	0.6161 (4)	0.0486 (8)	
H6	0.1789	0.8354	0.5112	0.058*	
C7	0.1464 (2)	0.7804 (3)	0.7451 (4)	0.0490 (9)	
H7	0.1628	0.7053	0.7247	0.059*	
C8	0.1310 (2)	1.1711 (3)	0.5473 (6)	0.0668 (10)	
H8A	0.1425	1.2130	0.4441	0.100*	
H8B	0.1706	1.1959	0.6356	0.100*	
H8C	0.0714	1.1843	0.5832	0.100*	
C9	0.1775 (3)	1.0189 (3)	0.3522 (5)	0.0838 (13)	
H9A	0.1816	1.0850	0.2805	0.126*	
H9B	0.1386	0.9645	0.3003	0.126*	
H9C	0.2349	0.9858	0.3656	0.126*	
C10	0.09047 (19)	0.6513 (2)	1.3124 (4)	0.0419 (7)	
C11	0.0228 (2)	0.6391 (2)	1.4323 (4)	0.0455 (7)	
C12	0.0272 (2)	0.5535 (2)	1.5526 (5)	0.0535 (8)	
H12	−0.0182	0.5450	1.6315	0.064*	
C13	0.0982 (2)	0.4804 (3)	1.5570 (5)	0.0613 (9)	
H13	0.1005	0.4228	1.6385	0.074*	
C14	0.1645 (2)	0.4925 (3)	1.4428 (5)	0.0600 (9)	
H14	0.2124	0.4433	1.4462	0.072*	
C15	0.1615 (2)	0.5780 (2)	1.3207 (5)	0.0522 (8)	
H15	0.2077	0.5861	1.2435	0.063*	
C16	−0.1075 (3)	0.7158 (4)	1.5491 (7)	0.1119 (18)	
H16A	−0.1517	0.7718	1.5246	0.168*	
H16B	−0.0789	0.7341	1.6553	0.168*	
H16C	−0.1345	0.6422	1.5578	0.168*	

### Atomic displacement parameters (Å^2^)

**Table:**

	*U*^11^	*U*^22^	*U*^33^	*U*^12^	*U*^13^	*U*^23^
N1	0.0510 (15)	0.0398 (15)	0.0465 (16)	−0.0020 (12)	0.0057 (14)	0.0021 (13)
N2	0.082 (2)	0.0495 (17)	0.0477 (19)	0.0023 (15)	0.0034 (16)	0.0051 (14)
O1	0.0611 (14)	0.0659 (14)	0.0565 (14)	0.0128 (11)	0.0183 (14)	0.0079 (13)
C1	0.0491 (18)	0.0401 (17)	0.056 (2)	−0.0014 (13)	0.0061 (17)	−0.0022 (18)
C2	0.0444 (16)	0.0412 (17)	0.047 (2)	−0.0059 (14)	0.0060 (16)	0.0013 (16)
C3	0.0525 (18)	0.0463 (17)	0.0506 (19)	0.0001 (14)	0.0084 (18)	−0.0012 (18)
C4	0.0532 (19)	0.0412 (17)	0.052 (2)	0.0033 (15)	−0.0015 (18)	−0.0010 (17)
C5	0.0472 (18)	0.0457 (18)	0.0393 (19)	−0.0050 (14)	−0.0056 (15)	0.0044 (16)
C6	0.0574 (19)	0.051 (2)	0.0379 (18)	−0.0011 (15)	0.0008 (15)	−0.0072 (16)
C7	0.057 (2)	0.0390 (18)	0.051 (2)	−0.0002 (15)	−0.0008 (16)	−0.0017 (16)
C8	0.072 (2)	0.051 (2)	0.077 (2)	0.0034 (17)	−0.005 (2)	0.0163 (19)
C9	0.123 (4)	0.070 (3)	0.058 (3)	−0.007 (2)	0.006 (3)	0.011 (2)
C10	0.0513 (18)	0.0315 (15)	0.0430 (18)	−0.0035 (14)	0.0018 (16)	−0.0028 (15)
C11	0.0547 (18)	0.0434 (17)	0.0383 (17)	−0.0013 (14)	0.0023 (17)	−0.0032 (16)
C12	0.061 (2)	0.0488 (19)	0.050 (2)	−0.0071 (16)	0.0132 (18)	0.0029 (17)
C13	0.076 (2)	0.0470 (19)	0.061 (2)	0.0001 (18)	−0.004 (2)	0.0091 (19)
C14	0.057 (2)	0.0491 (19)	0.074 (3)	0.0080 (15)	0.004 (2)	0.011 (2)
C15	0.0488 (18)	0.0497 (19)	0.058 (2)	−0.0004 (15)	0.0052 (17)	0.0025 (18)
C16	0.108 (3)	0.122 (4)	0.106 (4)	0.047 (3)	0.061 (3)	0.035 (3)

### Geometric parameters (Å, °)

**Table:**

N1—C1	1.272 (4)	C8—H8A	0.9600
N1—C10	1.423 (4)	C8—H8B	0.9600
N2—C5	1.356 (4)	C8—H8C	0.9600
N2—C9	1.425 (5)	C9—H9A	0.9600
N2—C8	1.450 (4)	C9—H9B	0.9600
O1—C11	1.370 (3)	C9—H9C	0.9600
O1—C16	1.390 (5)	C10—C15	1.382 (4)
C1—C2	1.453 (5)	C10—C11	1.398 (4)
C1—H1	0.9300	C11—C12	1.378 (4)
C2—C3	1.387 (4)	C12—C13	1.379 (4)
C2—C7	1.390 (4)	C12—H12	0.9300
C3—C4	1.374 (4)	C13—C14	1.353 (5)
C3—H3	0.9300	C13—H13	0.9300
C4—C5	1.410 (4)	C14—C15	1.386 (4)
C4—H4	0.9300	C14—H14	0.9300
C5—C6	1.401 (4)	C15—H15	0.9300
C6—C7	1.368 (4)	C16—H16A	0.9600
C6—H6	0.9300	C16—H16B	0.9600
C7—H7	0.9300	C16—H16C	0.9600
			
C1—N1—C10	118.4 (3)	H8B—C8—H8C	109.5
C5—N2—C9	120.9 (3)	N2—C9—H9A	109.5
C5—N2—C8	121.7 (3)	N2—C9—H9B	109.5
C9—N2—C8	117.2 (3)	H9A—C9—H9B	109.5
C11—O1—C16	117.3 (3)	N2—C9—H9C	109.5
N1—C1—C2	124.4 (3)	H9A—C9—H9C	109.5
N1—C1—H1	117.8	H9B—C9—H9C	109.5
C2—C1—H1	117.8	C15—C10—C11	118.6 (3)
C3—C2—C7	116.5 (3)	C15—C10—N1	122.8 (3)
C3—C2—C1	124.1 (3)	C11—C10—N1	118.6 (3)
C7—C2—C1	119.4 (3)	O1—C11—C12	124.4 (3)
C4—C3—C2	122.5 (3)	O1—C11—C10	115.8 (3)
C4—C3—H3	118.7	C12—C11—C10	119.7 (3)
C2—C3—H3	118.7	C11—C12—C13	120.7 (3)
C3—C4—C5	120.3 (3)	C11—C12—H12	119.7
C3—C4—H4	119.9	C13—C12—H12	119.7
C5—C4—H4	119.9	C14—C13—C12	120.0 (3)
N2—C5—C6	121.2 (3)	C14—C13—H13	120.0
N2—C5—C4	121.6 (3)	C12—C13—H13	120.0
C6—C5—C4	117.3 (3)	C13—C14—C15	120.3 (3)
C7—C6—C5	120.7 (3)	C13—C14—H14	119.8
C7—C6—H6	119.6	C15—C14—H14	119.8
C5—C6—H6	119.6	C10—C15—C14	120.7 (3)
C6—C7—C2	122.6 (3)	C10—C15—H15	119.6
C6—C7—H7	118.7	C14—C15—H15	119.6
C2—C7—H7	118.7	O1—C16—H16A	109.5
N2—C8—H8A	109.5	O1—C16—H16B	109.5
N2—C8—H8B	109.5	H16A—C16—H16B	109.5
H8A—C8—H8B	109.5	O1—C16—H16C	109.5
N2—C8—H8C	109.5	H16A—C16—H16C	109.5
H8A—C8—H8C	109.5	H16B—C16—H16C	109.5
			
C10—N1—C1—C2	−175.4 (3)	C1—C2—C7—C6	−177.0 (3)
N1—C1—C2—C3	−4.6 (5)	C1—N1—C10—C15	42.7 (4)
N1—C1—C2—C7	175.3 (3)	C1—N1—C10—C11	−140.4 (3)
C7—C2—C3—C4	−1.6 (5)	C16—O1—C11—C12	10.2 (5)
C1—C2—C3—C4	178.4 (3)	C16—O1—C11—C10	−170.9 (4)
C2—C3—C4—C5	−2.1 (5)	C15—C10—C11—O1	179.7 (3)
C9—N2—C5—C6	3.6 (5)	N1—C10—C11—O1	2.5 (4)
C8—N2—C5—C6	−170.9 (3)	C15—C10—C11—C12	−1.5 (4)
C9—N2—C5—C4	−175.8 (3)	N1—C10—C11—C12	−178.6 (3)
C8—N2—C5—C4	9.8 (5)	O1—C11—C12—C13	179.4 (3)
C3—C4—C5—N2	−176.2 (3)	C10—C11—C12—C13	0.7 (5)
C3—C4—C5—C6	4.5 (4)	C11—C12—C13—C14	0.2 (5)
N2—C5—C6—C7	177.4 (3)	C12—C13—C14—C15	−0.1 (5)
C4—C5—C6—C7	−3.2 (4)	C11—C10—C15—C14	1.5 (4)
C5—C6—C7—C2	−0.5 (5)	N1—C10—C15—C14	178.5 (3)
C3—C2—C7—C6	2.9 (5)	C13—C14—C15—C10	−0.7 (5)

### Hydrogen-bond geometry (Å, °)

**Table:**

*D*—H···*A*	*D*—H	H···*A*	*D*···*A*	*D*—H···*A*
C8—H8C···N1^i^	0.96	2.67	3.620 (5)	170
C4—H4···O1^i^	0.93	2.64	3.546 (4)	166

Symmetry codes: (i) −*x*, −*y*+2, *z*−1/2.

## References

[bb1] Atwood, D. A. & Harvey, M. J. (2001). *Chem. Rev.* **101**, 37–52. 10.1021/cr990008v 11712193

[bb2] Liu, X.-Y., Fan, Y.-H., Bi, C.-F., Wang, Q. & Gao, Y. (2009). *Acta Cryst.* E**65**, o2170. 10.1107/S1600536809031699 PMC2970039 21577576

[bb3] Sheldrick, G. M. (2008). *Acta Cryst.* A**64**, 112–122. 10.1107/S0108767307043930 18156677

[bb4] Siemens (1996). *SMART*, *SAINT* and *SADABS* Siemens Analytical X-ray Instruments Inc., Madison, Wisconsin, USA.

